# A Bibliometric Analysis of COVID-19 Publications Between January 2019 and February 2025 by Romanian Authors

**DOI:** 10.7759/cureus.82339

**Published:** 2025-04-15

**Authors:** Dragos Puia, Marius Ivanuta, Catalin Pricop

**Affiliations:** 1 Urology, University of Medicine and Pharmacy "Grigore T. Popa", Iași, ROU; 2 Urology, "CI Parhon" Clinical Hospital, Iași, ROU

**Keywords:** bibliometric analyses, coronavirus infectious disease, covid-19 outbreak, romanian population, sars-cov-2

## Abstract

The coronavirus disease 2019 (COVID-19) pandemic, declared by the World Health Organization (WHO) on March 11, 2020, had profound global consequences, including significant mortality, economic disruption, and strain on healthcare systems. Romania was also severely affected, with Suceava County being dubbed the "Romanian Lombardy" due to its high infection rates. In response, Romanian researchers actively contributed to scientific literature on COVID-19, producing numerous publications addressing epidemiology, public health policies, and medical treatments. This study aims to analyze Romanian scientific contributions related to COVID-19 using bibliometric methods.

A bibliometric analysis was conducted using Web of Science, focusing on publications from Romanian universities, hospitals, and medical organizations. Articles were selected based on relevance to medicine, while non-research publications such as editorials and book reviews were excluded. The study employed VOSviewer (available at https://www.vosviewer.com/) for co-authorship and keyword analysis, alongside CiteSpace (available at https://citespace.podia.com/) for citation burst analysis.

Between January 2019 and March 2025, 4,720 COVID-19-related articles with at least one Romanian author were indexed in Web of Science. After applying the inclusion criteria, 336 articles remained. The most productive authors were from Dunărea de Jos University, Galați, and the University of Oradea. Most studies were published in MDPI journals, with predominant research areas including internal medicine, pharmacology, and experimental medicine. Keyword analysis highlighted major research themes such as COVID-19 impact, severe acute respiratory syndrome coronavirus 2 (SARS-CoV-2), and pandemic response.

Romanian research significantly influenced public health policies, guiding pandemic management strategies. The crisis accelerated digital healthcare adoption, including telemedicine services. Biomarkers such as C-reactive protein (CRP), interleukin-6 (IL-6), and lactate dehydrogenase (LDH) were identified as predictors of COVID-19 severity. Additionally, the pandemic had severe psychological and social consequences, particularly among vulnerable populations. This bibliometric analysis underscores the substantial role of Romanian researchers in the global fight against COVID-19 and the lasting impact of their contributions. Understanding Romania’s scientific contribution to COVID-19 literature helps assess the country's research impact, identify strengths in key areas, and highlight opportunities for international collaboration in pandemic-related studies.

## Introduction and background

In December 2019, the World Health Organization (WHO) received reports of initial events of pneumonia of unknown origin, leading to the global spread of COVID-19, which was formally declared a pandemic by the WHO on March 11, 2020. The illness is induced by coronavirus, a substantial family of single-stranded RNA viruses. They are accountable for severe acute respiratory syndrome coronavirus (SARS) and Middle Eastern respiratory syndrome coronavirus (MERS) [1]. COVID-19 led to millions of deaths worldwide and caused significant strain on healthcare systems [2]. The pandemic also highlighted disparities in healthcare access and outcomes. The pandemic triggered extensive financial turmoil, resulting in unemployment, company failures, and a worldwide recession. Numerous nations instituted lockdowns and travel restrictions, exacerbating the economic effects [3].

The pandemic triggered an unprecedented surge in scientific research. Researchers worldwide focused on understanding the virus, developing vaccines, and finding treatments. The pandemic facilitated global cooperation among scientists, healthcare professionals, and governmental entities. This collaboration was essential for expediting vaccine development and facilitating the exchange of knowledge and resources. This led to a significant increase in publications related to COVID-19. According to Malekpour et al., between November 1, 2019, and April 15, 2021, 159132 papers were identified in PubMed and Scopus [4].

The pandemic also significantly impacted Romania. On March 16, 2020, Romania announced a "state of emergency" for 30 days. The most significant surge in COVID-19 cases during the initial wave of the pandemic occurred in Northwestern Romania, accounting for 47.1% of the infected medical personnel nationwide in April 2020. The adjacent Suceava County is frequently likened to the “Romanian Lombardy,” a poignant analogy to the Italian region significantly impacted by COVID-19 [5]. Researchers in Romania, similar to those worldwide, dedicated significant efforts to studying COVID-19, leading to a notable rise in scientific publications. Comparative studies indicate that Romania has a higher mortality rate than Greece, despite lower total case numbers, highlighting the impact of healthcare quality and vaccination rates [6]. This encompassed research on the virus's transmission, treatment, and effects on different facets of society. A substantial segment of the studies concentrated on public health strategies, epidemiological analyses, and the efficacy of treatments to mitigate the virus's transmission [6]. Prior to the pandemic, Romania had a developing but underrepresented presence in global biomedical research. As COVID-19 accelerated scientific output worldwide, Romania’s contributions grew, warranting a detailed bibliometric examination.

While Romania has contributed significantly to the global COVID-19 research effort, a structured analysis of its scientific output remains unexplored. Bibliometric analysis provides a valuable tool to assess research productivity, key focus areas, and collaboration trends, offering insights into Romania’s role in the global scientific landscape. Moreover, bibliometrics elucidates trends and deficiencies within the research domain, offering valuable insights into research priorities and prospects and guiding efforts toward areas necessitating advancement. VOSviewer (https://www.vosviewer.com/) and CiteSpace (https://citespace.podia.com/) are software tools designed for bibliometric analysis, helping researchers visualize networks and patterns within large collections of scientific literature. VOSviewer excels at creating clear maps showing relationships like co-authorship or keyword co-occurrence to identify research clusters. CiteSpace focuses more on analyzing the evolution of research fields over time, detecting emerging trends and identifying pivotal publications or turning points.

## Review

We conducted a comprehensive search in the Web of Science database using the keywords “COVID-19” or “SARS-CoV-2” to identify relevant publications. The bibliometric analysis was performed using the Web of Science Core Collection, selected for its comprehensive coverage of high-impact, peer-reviewed journals and structured citation data. Compared to other databases such as Scopus or PubMed, Web of Science provides advanced citation analysis tools and standardised indexing, making it particularly suitable for bibliometric evaluations. To refine the results, we applied specific filters, restricting the Region to “Romania” and including only studies affiliated with Romanian universities, hospitals, or other medical institutions. Each selected paper featured at least one Romanian author, ensuring the dataset reflected Romania’s contribution to COVID-19-related research. Additionally, we narrowed the scope to studies categorised under medicine as the primary research area, excluding articles from unrelated disciplines. We used the "Research area" filter and selected all fields related to Medicine (e.g., General Internal Medicine, Infectious Diseases, Immunology, and so on). Fields unrelated to medicine (e.g., business economics, computer science, physical geography) have been excluded. The Web of Science data was saved as a .txt file and imported into the software at the end of the search. The maximum number of authors per document was 25, and the minimum number of authors per record was three.

Following the initial selection, we implemented a set of exclusion criteria to enhance the focus of our analysis. We systematically removed meeting abstracts, editorial content, correspondence, notes, errata, early access publications, news articles, and book reviews, as these document types do not typically contribute to original research or substantial scientific findings. This filtration allowed us to concentrate on peer-reviewed studies that provided significant insights into the medical and scientific dimensions of COVID-19 research in Romania. No manual screening or risk of bias assessment was applied, as this study aimed to map the publication landscape rather than evaluate individual study quality.

For the bibliometric analysis, we employed VOSviewer 1.6.18 (Centre for Science and Technology Studies, Leiden University, The Netherlands, available at https://www.vosviewer.com/), a specialised software tool for visualizing and analyzing bibliometric networks. The analysis encompassed multiple aspects, including author collaboration networks, institutional affiliations, national contributions, journal impact, cited journals, cited authors, and keyword co-occurrence patterns. Furthermore, CiteSpace software (available at https://citespace.podia.com/) was utilised to identify and analyse references exhibiting citation bursts, highlighting research topics that gained significant traction over time.

To explore collaborative trends, we performed co-authorship analysis at both the individual and institutional levels, revealing interconnections between researchers and organisations. Additionally, we conducted a co-occurrence analysis of keywords, providing insights into dominant research themes and emerging topics. The network visualisations generated by VOSviewer represented these relationships using cluster maps, where the size of each node corresponded to the volume of associated research output. Nodes were assigned distinct colours to indicate different clusters, facilitating the identification of major thematic groups.

The authors manually assigned cluster labels in the keyword cluster map by reviewing high-frequency terms within each group to enhance interpretability. This step ensured that the thematic categorisations accurately reflected the underlying research trends. The resulting analysis offers a structured overview of Romania’s scientific engagement with COVID-19, highlighting key contributors, influential studies, and prevailing research directions.

**Figure 1:**
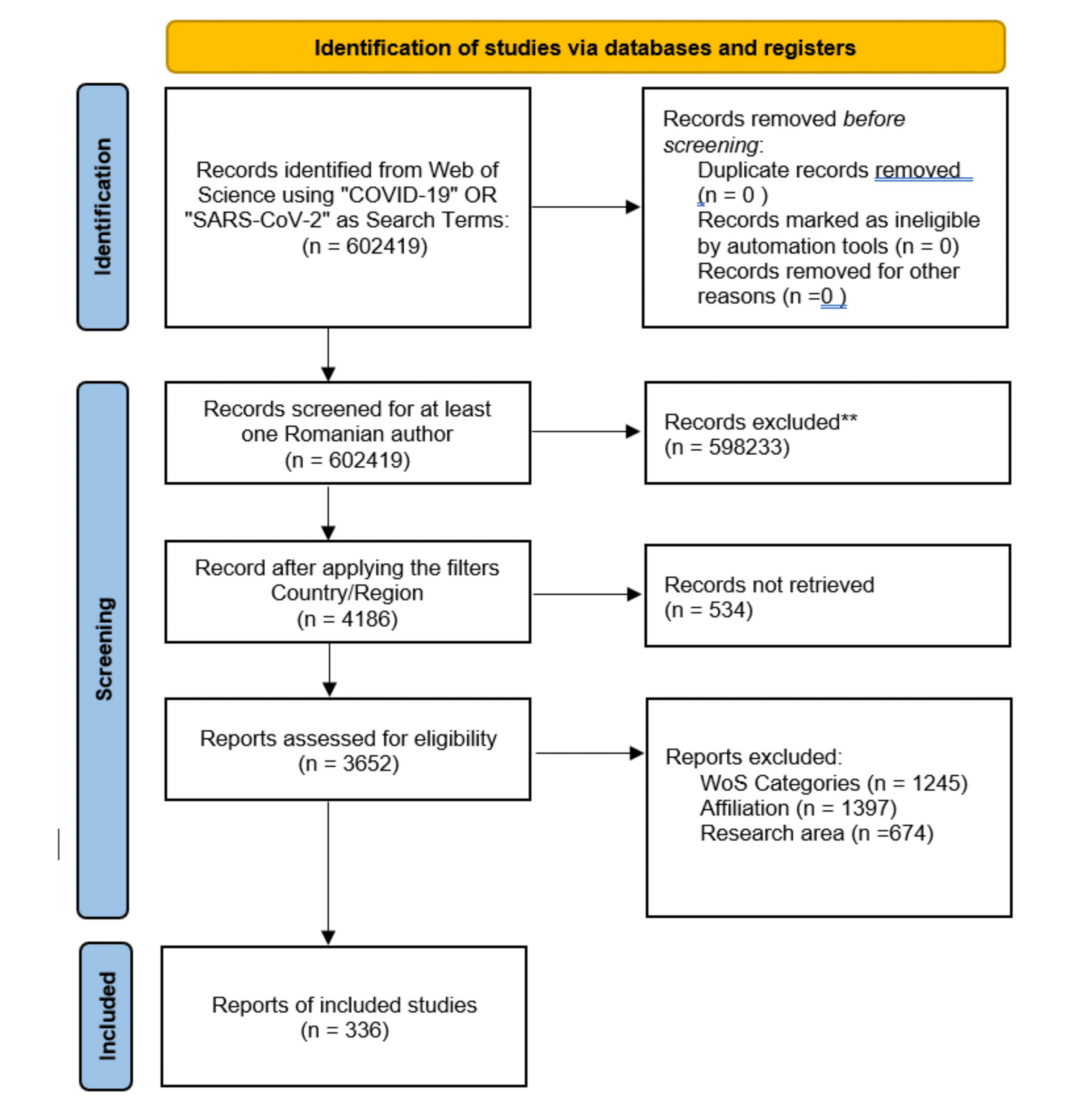
Study selection process for included articles

Between January 2019 and February 2025, the Web of Science indexed 4720 articles on COVID-19 or “SARS-CoV-2” with at least one Romanian author. After applying the filters Country: Romania and Affiliation: Romanian universities, hospitals, or other medical organisations, 4186 documents remained. After further refining the research area, only 336 papers were included. Figure 1 represents the flowchart of the study screening and selection process.

The top 10 most productive authors are listed in Table 1. 

**Table 1:** Leading Romanian authors in COVID-19 research: publications and citations overview

	Author	Affiliation	Documents	Citations
1	Ciubara Anamaria	Dunarea de Jos University, Galaţi	19	106
2	Nechita Aurel	Dunarea de Jos University, Galaţi	16	96
3	Bungau Simona	University of Oradea	16	402
4	Bratosin Felix	Victor Babeș University of Medicine and Pharmacy Timișoara	13	64
5	Tatu Alin Laurentiu	Dunarea de Jos University, Galaţi	13	72
6	Baroiu Liliana	Dunarea de Jos University, Galaţi	12	125
7	Anghel Lucretia	Dunarea de Jos University, Galaţi	11	129
8	Birlutiu Victoria	Lucian Blaga University of Sibiu	11	90
9	Lupu Vasile Valeriu	Grigore T Popa University of Medicine and Pharmacy, Iaşi	10	39
10	Miftode Ionela Larisa	Grigore T Popa University of Medicine and Pharmacy, Iaşi	10	105

**Figure 2:**
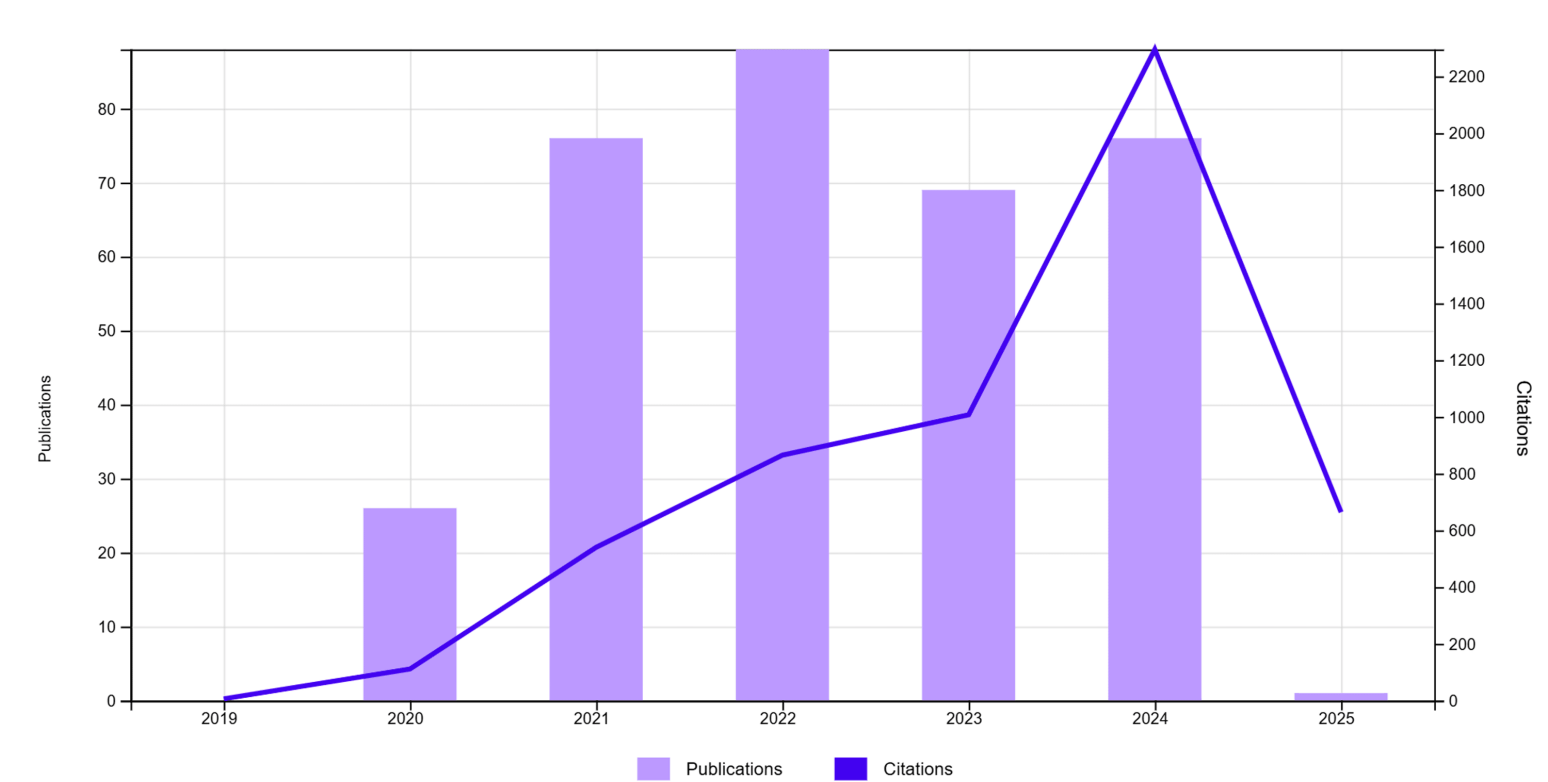
Visualisation of publication and citation each year

The number of documents published each year and the citations are represented in Figure 2.

**Figure 3:**
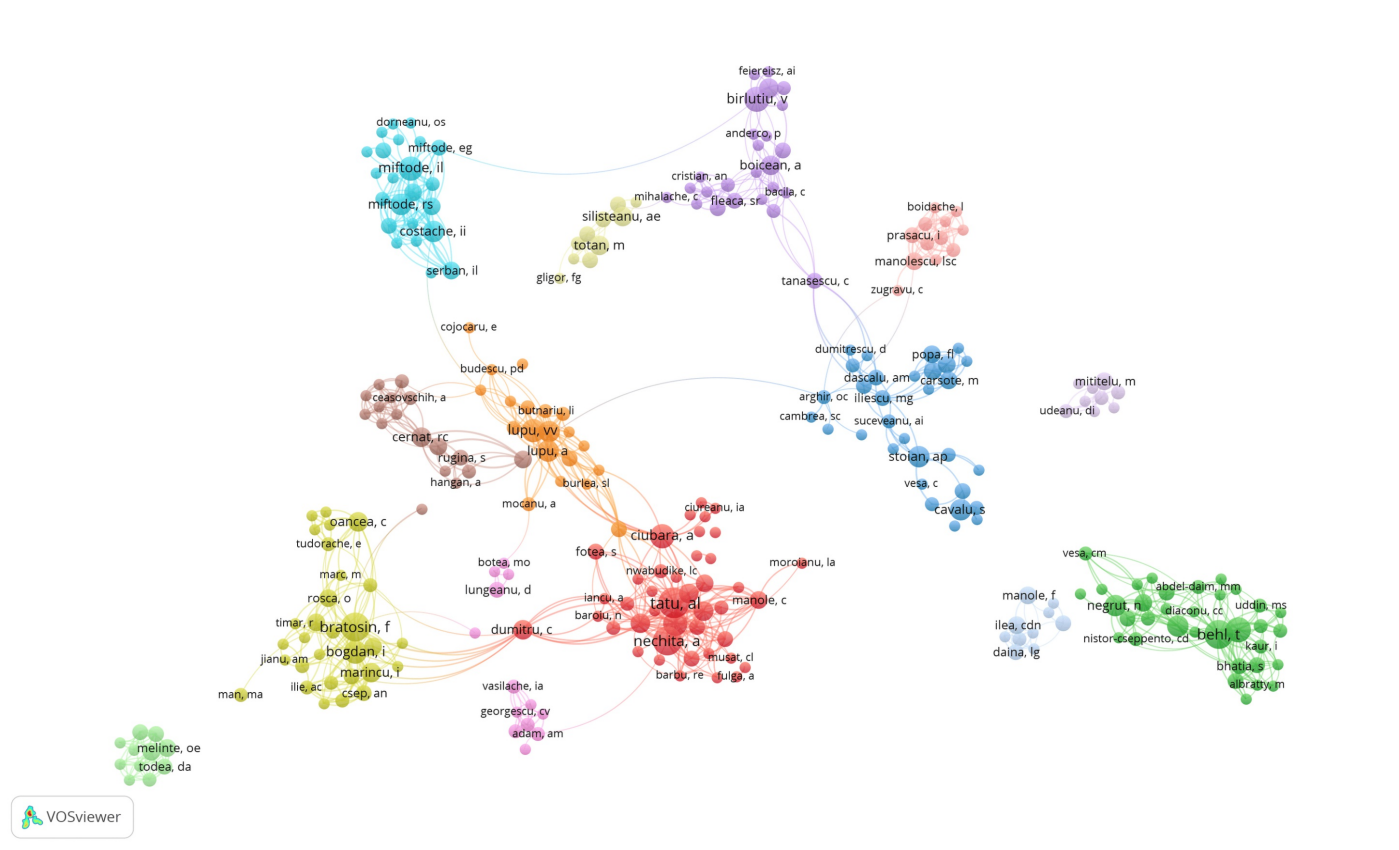
Visualisation collaborative networks among authors The image is generated by the authors using the VOSviewer software (available at https://www.vosviewer.com/). The image reflects the visualization of data processed from the relevant literature according to the chosen keywords.

Regarding the Web of Science categories, most of them targeted areas such as general internal medicine (31.718%), pharmacology & pharmacy (17.621%), experimental medicine (16.300%), health care sciences services (12.335%), immunology (8.370%), and the rest in other topics. The collaboration between authors is shown in Figure 3.

The first five publishers in whose journals the articles were published were represented by MDPI (44.94%), Elsevier (8.92%), Springer Nature (5.65%), Frontiers Media Sa (5.35%) and Edusoft Publishing (3.86%). Considering that the publisher with the most articles was MDPI, the top five journals it was published most frequently were all from the same publisher, respectively: Journal of Clinical Medicine (8.81%), Healthcare (7.04%), Journal of Personalized Medicine (4.84%), Medicina Lithuania (4.84%), and Biomedicines (4.40%).

**Figure 4:**
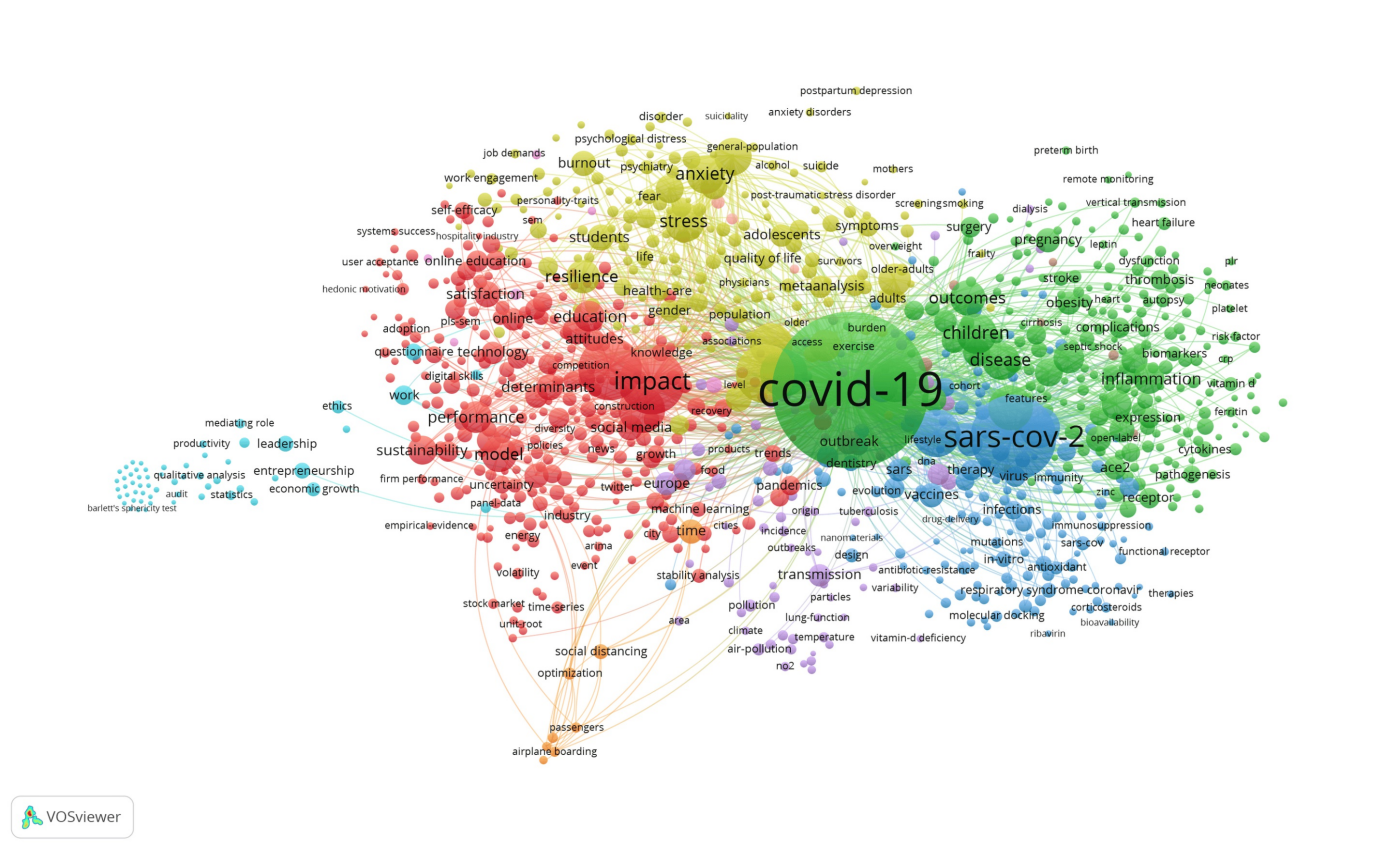
Visualisation of keywords clusters The image is generated by the authors using the VOSviewer software (available at https://www.vosviewer.com/). The image reflects the visualization of data processed from the relevant literature according to the chosen keywords.

Visualisation was conducted following the consolidation of duplicate keywords and setting a minimum occurrence threshold of three for each keyword. These keywords were automatically categorised into four primary and three secondary clusters in VOSviewer, emphasising the most frequently used: COVID-19, Sars-Cov-2, impact, and pandemic, as shown in Figure 4.

The impact of Romanian scientific literature on public health policy during the COVID-19 crisis was substantial, as it furnished critical data and ideas that informed official actions. Incorporating scientific evidence into policy-making enabled a better-informed strategy for pandemic management, affecting several areas such as healthcare, business, and education. According to Persu MR, the pandemic had a significant demographic impact [7]. The mortality rate was notably high among individuals over 60 years of age, which significantly increased the pressure on the healthcare system. This demographic was particularly vulnerable to severe outcomes from the virus, leading to a higher number of deaths in this age group [7]. Also, the authors highlighted the disproportionate impact of COVID-19 on elderly populations, influencing healthcare resource allocation. The epidemic imposed enormous pressure on Romania's healthcare system. The high mortality rates and increasing demand for medical services during the State of Emergency and Alert exposed the system's shortcomings and the need for changes in healthcare infrastructure.

As a result, chronic patients' therapeutic plans have been modified. In the case of oncology patients, it affected various aspects of their treatment and overall well-being. Many cancer patients experienced delays in their treatment due to the pandemic. Hospitals and clinics were overwhelmed with COVID-19 cases, leading to postponed surgeries, chemotherapy, and radiotherapy sessions. The pandemic led to an increase in mortality rates among cancer patients. Those who contracted COVID-19 had a higher risk of severe complications and death, especially if they were undergoing immunosuppressive treatments [8,9].

On the other hand, the pandemic has accelerated the adoption of new medical technologies. According to Brînzac, one of the most notable achievements is establishing telemedicine services for family doctors. Initially introduced during the lockdown to curb the spread of the virus, these services were extended due to advocacy from professional associations and civil society [10]. In addition, studies by Brînzac et al. contributed to policy changes that extended telemedicine services beyond the lockdown period [10]. This shift has improved access to healthcare for many patients, especially during challenging times. The epidemic accelerated Romania's public health sector's digitisation. It emphasised digital solutions and increased technology and service adoption [10].

Romanian scientific literature has contributed to worldwide awareness of the COVID-19 pandemic through literary reflections and epidemiological analysis. Romanian authors and scholars have contributed to the global discussion on the pandemic's effects on society, health, and transmission dynamics. According to Musat et al., in Romanian patients, biomarkers have been critical in understanding the severity and progression of COVID-19. Specific markers such as CRP, IL-6, LDH, and others have been associated with increased severity, risk of Intensive Care Unit admission, and mortality, highlighting their potential utility in clinical practice [11]. Results consistent with those of other authors [12,13]. Mitrea et al. identified that a significant portion of the Romanian population experienced psychological distress during the pandemic [14]. The authors showed that individuals who were unemployed or retired reported significantly higher levels of anxiety compared to those employed in other sectors.

The COVID-19 pandemic has resulted in an exponential growth in scientific publications. By the end of the first two years of the pandemic, the number of COVID-19-related publications had already surpassed the total of publications on previous coronaviruses such as SARS, MERS, H1N1, and Ebola. The number of publications related to COVID-19 has surged, with a significant increase in research output observed globally. For instance, infodemic research doubled post-pandemic onset, with the United States and China as major contributors [15]. The United States and China have been the most active countries in publication output, with the US leading in non-pharmaceutical interventions, treatment, and vaccine-related research. At the same time, China has focused more on clinical features, virology, and epidemiology [16]. According to Wang et al., universities such as Harvard University, the University of Oxford, and the University of Toronto have been at the forefront of COVID-19 research, particularly in areas such as vaccine development and clinical studies [16]. Low- and middle-income countries have been underrepresented in COVID-19 research, reflecting broader disparities in scientific capacity [17]. While the bibliometric analysis highlights the extensive research efforts and collaborations, it also underscores the challenges in research quality and the need for more robust studies. The findings suggest potential areas for future research, such as conducting double-blinded randomised controlled trials to validate existing findings and further exploring the long-term impacts of COVID-19 on various sectors [18].

Romania's contributions to understanding COVID-19 spanned several areas, although perhaps without the level of high-profile, globally disruptive discoveries seen in some larger research nations. According to Capraru et al., there was a disproportionate impact of COVID-19 on Roma communities in Western Romania. The authors highlighted the importance of personalized care strategies for marginalized populations [19]. Romanian authors revealed how the pandemic accelerated the adoption of telemedicine solutions in Romania, with efforts to develop integrated e-health systems. Some companies launched innovative digital projects, such as online payment solutions for meal cards and partnerships to facilitate online bookings, in response to pandemic challenges. Restaurants also adopted online food ordering and delivery platforms [20].

Our work, however, also has some limitations. The study is limited to the Web of Science database, which may exclude relevant research published in other indexing platforms like Scopus, PubMed, or Google Scholar. The motivation for this situation stems from the fact that many search filters are available on the Web of Science, and our goal was to limit ourselves to Romanian authors. While our study presents citation counts, it does not provide insights into the quality or impact of highly cited papers in the field. Last but not least, the keyword clustering approach might be influenced by variations in terminology, potentially missing key themes due to differences in how different authors use terms.

## Conclusions

Romanian scholars' bibliometric examination of COVID-19 material underscores Romania's substantial scientific contribution to comprehending and managing the epidemic. Collaboration networks among Romanian researchers were illustrated, highlighting the significance of institutional collaborations in promoting COVID-19 research. The results highlight Romanian scientific literature's influence on public health policy establishment, especially on pandemic response tactics. The analysis indicates a transition to digital healthcare solutions, including telemedicine, as a lasting consequence of the crisis. The research illustrates the pandemic's impact on several medical disciplines, including oncology and biomarker-driven illness evaluation. The study offers insights into Romania's scientific scene during the epidemic and its contributions to world knowledge.
